# Severity of Binge Eating Behavior among Overweight College Students in Taiwan and Associated Factors

**DOI:** 10.3390/healthcare11030338

**Published:** 2023-01-24

**Authors:** Huey-Yeu Yan, Chieh-Yu Liu, Mei-Chih Meg Tseng, Tzu-Ying Lee, Pei-Fan Mu, Hung-Ru Lin

**Affiliations:** 1 School of Nursing, National Taipei University of Nursing and Health Sciences, Taipei City 112303, Taiwan; 2 Department of Nursing, University of Kang Ning, Taipei City 114311, Taiwan; 3 Department of Health Care Management, National Taipei University of Nursing and Health Sciences, Taipei City 112303, Taiwan; 4 Department of Psychiatry, School of Medicine, College of Medicine, Taipei Medical University, Taipei City 110301, Taiwan; 5 Department of Psychiatry, Shuang Ho Hospital, Taipei Medical University, New Taipei City 235041, Taiwan; 6 School of Nursing, National Yang Ming Chiao Tung University, Taipei City 112304, Taiwan

**Keywords:** binge eating, overweight, obesity, college students, severity

## Abstract

Background: Binge eating (BE) is considered a marker of obesity and overweight and a significant characteristic of feeding and eating disorders. Despite the high prevalence of obesity on college campuses, the issue of BE among college students in Taiwan has received little attention. The aim of this study was to investigate BE behavior among overweight college students in Taiwan and associated factors. Methods: This study utilized a cross-sectional survey. A total of 300 overweight college students were recruited through convenience sampling. Data were collected using a self-administered Binge Eating Scale (BES) and a body weight composition monitor (Model No. OMRON, HBF-126) and analyzed using descriptive statistics, correlation analysis, and regression analysis. Results: The average BES score was 10.67 (SD = 6.66, 0–34). With a BES score of 17 as the cut-off point, 17.3% (n = 52) of the participants were found to have moderate or severe BE behavior. Analysis of the demographic and psychosocial data using Spearman’s rho rank correlation coefficient revealed that sex, body mass index (BMI), uncontrolled eating, weight loss diets, academic stress, peer competition, interpersonal distress, and unpleasant or major life events were significantly correlated with BE behavior and its probability (r_s_ = −0.14–0.15, *p* < 0.05). Furthermore, logistic regression analysis indicated that the odds ratio of the BES scores of female participants and those who stated to have experienced uncontrolled eating, weight loss diets, peer competition, and interpersonal distress was 1.05–6.04 times those of male participants and those without such experiences (*p* < 0.05). Conclusion: The study found that nearly one-fifth of participants presented moderate to severe levels of BE behaviors, and these were significantly correlated with sex and external environmental stress. This study suggests early intervention from campus psychological health personnel to provide proper therapy.

## 1. Introduction

Obesity is one of the most common health issues experienced by college students, and this situation has not improved in the 21st century. According to the data reported by the American College Health Association [1], over one-third of college students (n = 80, 121) are overweight (21.6%; 25.0 ≤ BMI < 30.0) or obese (12.5%; BMI ≥ 30.0). A similar situation exists in Taiwan, with one government report revealing that the prevalence of overweight and obesity in the 18~24-year-old college demographic has been considerably high (15.3% and 14.0%, respectively) in recent years (2016–2019) [2]. Given the high proportion of obesity in the college student population, this issue requires immediate attention.

Binge eating (BE) is considered a marker of obesity and weight gain, and a significant characteristic of feeding and eating disorders [3]. Research studies have revealed that its prevalence among college students is 7.3~13.5% [4,5,6], even escalating to 17~34.9% in overweight and obese subgroups [7,8], which is much higher than in the general population (1~3%) [9,10]. Additionally, BE has a population prevalence of about 3.5% in women and 2.0% in men [5,6,11,12]. Based on the Diagnostic and Statistical Manual of Mental Disorders, Fifth Edition (DSM-5), released by APA (American Psychiatric Association) in 2013, the definition of BE is eating an excessive amount of food in a short period of time (usually within 2 h), accompanied by a feeling of loss of control. During most binge episodes, at least three of the following behaviors emerge: eating rapidly, eating until uncomfortably full, eating while not hungry, eating alone due to embarrassment, and feeling disgusted, depressed, or guilty after overeating [13]. Therefore, BE could cause excessive calorie intake and weight gain, which, in turn, increases the risks of heart disease, type 2 diabetes, digestive problems, and psychological distress over binging, eventually harming physical and mental health [14,15].

The cause of BE is complicated and remains unclear. However, research studies have indicated that victims of teasing or bullying due to their body shape are 2–3 times more likely to binge eat than those without such experiences [16,17]. Additionally, over-dieting and being overly self-conscious about body weight are also considered to have a significant correlation with BE behaviors [3,18,19,20]. A study exploring the prevalence of binge eating, psychiatric comorbidity, and academic performance in first-year college students (n = 4889) indicated that body weight is significantly correlated with daily meal frequency and stress felt (*p* < 0.05). Furthermore, academic performance is also significantly correlated to the incidence of BE: typically, lower academic performance (−4.1 to −11.2% range) and higher risk of academic failure (ORs = 1.4–4.2) are related to a higher risk of BE incidence [5].

Furthermore, interpersonal relationships could be another factor associated with BE. Ferguson et al. [21] studied the influences of TV, social media, and peer pressure on young female students’ (n = 237) body dissatisfaction, eating disorder symptoms, and life satisfaction and revealed that peer competition is the most effective predictor for eating disorders, especially peers’ negative language about body weight causing mental pressure and negative emotions in the victim. Additionally, some studies have pointed out that individuals’ negative experiences and intrafamily relationships, such as sexual assault, domestic violence, and parental divorce, also have considerable correlations with BE behaviors [20,22].

Based on the research findings above, BE is closely associated with body weight and emotional distress. However, official policies to address obesity issues on campus still predominantly focus on traditional measures such as restricting dietary intake, reducing calorie intake, and increasing activity. For example, the Health Promotion Administration (HPA) has advised a daily calorie intake of less than 1200 calories and at least 300 min of medium-level physical activities weekly [23]. Very little research has been conducted on BE thus far. Only a few studies have been performed, including some on bulimia nervosa [24,25,26,27] and one on the correlation between weight stigma and BE behaviors [28]. Therefore, this study aims to investigate the severity and related factors of BE behavior among college students in Taiwan. The research hypothesis is that specific demographic variables (sex, weight) and psychosocial variables (uncontrollable eating, weight loss diets, being ridiculed for body shape, academic stress, peer competition, family pressure, interpersonal distress, and unpleasant/major life events) are significantly correlated with BE behaviors. Hopefully, this study can serve as a reference for campus health administrations in developing mental health interventions in the future.

## 2. Materials and Methods

### 2.1. Participants and Procedure

Once the study was reviewed and approved by the Institutional Review Board (TCHIRB-10910001-E), 300 overweight and obese college students were recruited from five Taiwanese universities via convenience sampling. The overweight standards posted by the Health Promotion Administration in Taiwan [23], including (a) body mass index (BMI) ≥ 24 kg/m^2^ for overweight and (b) age between 18 and 24, were adopted as the inclusion criteria. The exclusion criteria were (a) being pregnant, (b) taking antipsychotics, and (c) refusing to participate or sign the consent form. The number of samples was determined according to the literature, which was calculated by multiplying the number of questions (16 major questions and 62 sub-questions, each with 3–4 options) by 3–5 [29,30,31]. G*power 3.1.9.4 was also used with the following input parameters: odds ratio = 2.5, Ho = 0.15, α = 0.05, and power = 0.8 for estimation. An attrition rate of twenty was considered [32]. 

The survey was conducted using a face-to-face approach. Prior to data collection, researchers first approached potential participants on the college campus to explore their interest in participating by explaining the purpose and procedure of the study. If they were interested, fully understood the research content, and signed the consent form, the survey was carried out. During data acquisition, a body fat monitor was used to automatically calculate the participant’s BMI based on real-time weight measurements and college entrance PE (physical examination) height data. Participation was voluntary. The questionnaires were self-administered and the data were kept anonymous. Anyone who refused to take part was excluded from data collection. The study invited 335 potential participants, and 310 of them were willing to take part. After further excluding 10 people who did not meet the inclusion criteria (BMI < 24), a total of 300 participants were recruited for the study.

### 2.2. Measurements

The study tools included a psychosocial demographic datasheet, a BES questionnaire, and a body fat monitor. 

#### 2.2.1. Demographic and Psychosocial Data

Participants were asked to input their age, and experience of previous events including uncontrolled eating, weight loss diets, being ridiculed for body shape, academic stress, peer competition, family pressure, interpersonal distress, and unpleasant/major life events. In the assessment of the experience of previous events, participants were asked to respond with Yes or No. The definition of uncontrolled eating is consuming a large amount of food in a short time with a sense of losing control. Weight loss diet means overly restraining from eating for the sake of weight loss. Being ridiculed for body shape means his/her body shape is laughed at by others. Academic stress means stress from academic requirements in school. Peer competition means competition with peers in all ways. Family pressure means pressure from intra-family problems. Interpersonal distress means having difficulty in interacting with others. Unpleasant/major life events refer to traumatic events in one’s life. For the major life events, participants were asked open questions. On the other hand, their body height and weight were collected from the college entrance physical examination report, and BMI was calculated by a body weight composition monitor (Model No. OMRON, HBF-126).

#### 2.2.2. Body Mass Index (BMI)

BMI was calculated and automatically displayed on the screen using the standard formula (BMI = weight (kg)/height^2^ (meter^2^)), based on the height data registered in the body fat monitor and weight measured in real time.

#### 2.2.3. Binge Eating Scale (BES)

The purpose of the BES, developed by American psychiatrists [33], is to assess the severity of BE behavior among obese individuals. It comprises two major components: (1) eating behaviors (8 questions), such as rapid eating or eating when not physically hungry, and (2) emotional cognition upon BE (8 questions), such as feeling guilt or self-hate after overeating and feeling helpless about controlling eating. Each question has three or four options and is scored 0–2 or 0–3, respectively. A total score is then calculated (ranging from 0 to 46). The higher the total score, the more severe the BE problem.

The BES has suitable psychometric properties, with an internal consistency of Cronbach’s α > 0.8 [34,35,36]. When the boundary is divided into three score ranges to distinguish the severity—i.e., ≤17 = none to mild, 18-26 = moderate, and ≥27 = severe BE problem—the discriminability of the questionnaire reaches 96.7% [37,38]. The BES has been translated into multiple languages and applied to different ethnic groups, including overweight and obese college students [19,34,37,39]. After obtaining the original author’s consent, the research team translated the questionnaire into Chinese. After that, two professionals performed back translation to English, and the original author, Gormally, was invited to check the equivalence in terms of semantics and text. Seven experts were invited to review its content validity using a four-point scale (scale level CVI = 0.99). The confirmatory factor analysis showed a reasonable model fit (GFI = 0.91, AGFI = 0.89, RMSEA = 0.06), which was consistent with the original BES. Internal consistency reliability and the intraclass correlation coefficient (ICC) of repeated measures apart were both 0.83, indicating cross-cultural adaptation.

### 2.3. Data Analysis

Data analysis was carried out using SPSS 22.0 statistical software. Participant characteristics were analyzed by descriptive statistics, including percentage, frequency, mean, and standard deviation. An independent samples *t*-test was adopted to compare behavioral and emotional cognition scores between the two groups (with and without BE problems). Additionally, the relationship between BES score and the variables body weight, sex, uncontrollable eating, weight loss diets, ridiculed body shape, academic stress, peer competition, family pressure, interpersonal distress, and unpleasant/major life events were examined by correlation analysis. Furthermore, logistic regression analysis was used to determine the predictive value of BE risk and evaluated by the odds ratio (OR).

## 3. Results

### 3.1. Participant Characteristics

Of the 300 participants, the average age was 20.37 years, the average BMI was 29.82 kg/m^2^, and most participants were women. Among them, 15.3% reported experiencing uncontrolled eating, and at least half had experienced a weight loss diet, being ridiculed for body shape, and academic stress. Additionally, some participants expressed that they had experienced peer competition (31.7%), family pressure, interpersonal distress, and unpleasant/major life events. The average BES score was 10.67. Among the 300 participants, 248 had mild or no BE problems, 46 had moderate BE problems, and 6 had severe BE problems (Table 1).

### 3.2. Binge Eating Behavior

According to the participants’ responses to the BES (Table 2), in terms of eating behavior (Q2, 4, 5, 7, 8, 9, 11, and 13), most participants ate quickly and felt uncomfortably full afterward (75.7%), and 7.0% used to eat when feeling bored. Furthermore, more than half of the participants felt the urge to eat (61.0%) even if they were not hungry, a considerable amount lost control of their eating while on a diet (38.3%), and many would eat too much food on a regular basis (68.3%). The results also suggested that many participants’ calorie intake fluctuated significantly (67.7%), with more than half of them not being able to stop eating even when full (57.7%), and a few used to eat between meals (9.3%).

Alternatively, in terms of emotional cognition upon an episode of BE (Q1, 3, 6, 10, 12, 14, 15, and 16), many of the participants were concerned with their appearance or body weight (37.7%); a similar number of them felt powerless in controlling their diet (32.7%); many felt guilty after overeating (67.7%); a considerable number could not control their desire to eat (34.7%); and a number of them felt embarrassed when eating in front of people (36.0%). Many participants also mentioned that their thoughts were often occupied by the desire to control their diet (63.7%), more than half consistently had strong food cravings (53.0%), and a fair amount sensed their physical hunger and ate a proper amount of food (36.7%).

Subsequently, an independent samples t-test was adopted to compare behavioral and emotional cognition scores between participants with (n = 52) and without (n = 248) BE. The results revealed significant differences between the mean score of the above 2 factors (11.4 ± 2.6 vs. 4.7 ± 2.7; 10.6 ± 2.7 vs. 3.6 ± 2.6, respectively, *p* < 0.01). Therefore, the total BES mean scores also presented a significant difference, 21.9 ± 3.6 vs. 8.3 ± 4.3 (*p* < 0.01).

### 3.3. Correlation between BE Behavior and Demographic or Psychosocial Characteristics

Since the data were not normally distributed, Spearman’s rho rank correlation coefficient was adopted to explore the correlation between the BES score and demographic characteristics. The results indicated that BE behavior was significantly correlated with sex, height, and BMI (r_s_ = −0.14, −0.13, and 0.12, respectively, *p* < 0.05), whereas its correlations with age and weight failed to reach statistical significance (r_s_ = −0.02 and 0.04, respectively, *p* > 0.05). Alternatively, in terms of psychosocial characteristics, the probability of BE was significantly correlated with self-reported experiences of uncontrolled eating (r_s_ = 0.40), weight loss diets (r_s_ = 0.20), being ridiculed for body shape (r_s_ = 0.20), academic stress (r_s_ = 0.17), peer competition (r_s_ = 0.23), interpersonal distress (r_s_ = 0.23), and unpleasant/major life events (r_s_ = 0.15; *p* < 0.01, for all). The only exception among psychosocial characteristics was family stress (r_s_ = 0.09, *p* > 0.05).

Additionally, logistic regression analysis was used to predict risk. After significant variables such as sex, uncontrolled eating, weight loss diets, peer competition, and interpersonal distress were controlled, the bivariate analysis suggested that the odds ratio (OR) of the BES score of female participants was 1.05 times that of male participants (*p* = 0.02). The results also indicated that, in the case of participants with reported experiences of uncontrolled eating, weight loss diets, peer competition, and interpersonal distress, their BES scores were higher than those without such experiences (Table 3).

## 4. Discussions

BE has been reported as a common eating disorder for overweight college students in Western countries. Due to the increasing rate of overweight among college students (18–24-year-olds) in Taiwan, the relationship between BE and obesity demands further exploration. This study utilized a BES questionnaire (cut-off score = 17) to evaluate the severity and associated influencing factors for BE behaviors among overweight college students in Taiwan. It was found that 17.3% of participants have medium to severe BE problems, at the low end of the research data found in Western countries (17–34.9%) [7,8]. It is reasonable to estimate that BE behaviors could be caused by complicated interactions between physical, mental, and environmental conditions and that the incidence of BE is related to ethnic and cultural backgrounds [40,41]. Biological and environmental factors should be considered in future studies. This study represents a rare effort in today’s Taiwanese research domain: it focused on overweight and obese college students and studied their BE behaviors. Our findings could enhance clinical empirical data and serve as a supportive reference for college campus health personnel.

To date, only a few studies have been performed on overweight or obese college students’ BE issues in Taiwan. Compared to Tseng et al. [42], it appears that the prevalence of BE in this study is higher. They found that out of the 189 adults who participated in a hospital weight loss program, 15.9% (n = 30) suffered from BE. However, compared to Wu and Liu [28], who reported a prevalence of 19.9% (n = 28) among 141 overweight adults recruited from a hospital for weight stigma and BE research, the prevalence in our study was lower. In addition to the differences between the two participant groups, a possible explanation could be that the former utilized the Bulimic Investigatory Test, Edinburgh to assess the BE behavior of participants (with a critical value ≥20), which is highly sensitive for the diagnosis of BE and frequently used for clinical treatment and monitoring to obtain a lower prevalence. Alternatively, while the latter also adopted BES for assessment, participants who were (mostly) obese and susceptible to BE and already seeking treatment were recruited from medical centers. Therefore, both studies reported a prevalence of BE different from the results of this study. Due to cultural and economic environmental influences in Asia, eating disorders, particularly in younger people, have historically lacked attention. Clinical research also indicates that a lack of medical attention information and medical resources might be the main reason [43]. Furthermore, people exhibiting BE behaviors tend to hide their problems [28]. It is recommended that future studies conduct comprehensive investigations focusing on college students’ eating problems, so as to identify those at high risk of BE and to provide necessary help at an early stage.

Demographic data analysis in this study revealed that BE behaviors are significantly correlated with sex and BMI, with women presenting a higher BE risk than men. Furthermore, BE risk increases as BMI does. This finding is consistent with specific reports in Western countries [5,9,10,12,39]. It is estimated that a slim figure is still an important element of beauty in both Eastern and Western societies, even with the evolution of the times. On top of that, traditional and social media as well as commercials promoting slimness are everywhere. Women have been made to feel concerned about their body shape, thus making them prone to eating disorders [44,45,46]. It is recommended that future studies focus on body dissatisfaction and sociocultural factors to further understand their connection with eating psychopathology. On the other hand, this study revealed little or no significance for age and body weight variations; this may be related to the small age variation among participants and using BMI as the only indicator of obesity. It is recommended for future research to employ a larger sample size and more indicators of obesity such as body fat percentage, skin-fold thickness, and waist-to-hip ratio to establish a baseline of the norm. Since the risk of BE increases with increasing BMI, campus health personnel should acquire and analyze students’ BMI data to help identify a high-risk group for BE and provide an intervention and support.

In addition, psychosocial analysis has indicated that the probability of BE is significantly correlated with uncontrolled eating, weight loss diets, interpersonal distress, and being ridiculed for body shape, which is consistent with the findings of multiple similar studies in Western countries [16,18,19,20]. Owing to the significant correlation between the occurrence of eating disorders and rigorous dieting regimes as well as ridiculed body shape [17], college health organizations should provide weight control programs and dietary as well as mental health therapeutic interventions for individuals to establish an appropriate weight loss program and to increase satisfaction with self-image. Peer pressure, interpersonal distress, academic stress, and unpleasant/major life events are also significantly correlated with BE behaviors, and this finding is consistent with the literature in Western countries [5,21]. Although family pressure is not significantly correlated with BE behaviors, one study of Taiwanese college students reported that both lacking and excessive parental care are significantly correlated with BE behaviors [47]. It is recommended to expand the diversity of the sample and to focus on such variables in future studies. Lastly, the results of this study support that external environmental stress has an important relationship with the BE behaviors of overweight college students. It is recommended that relevant units in schools develop courses for stress and emotion management to help individuals learn positive coping techniques.

### Limitations

Despite being one of the few studies focused solely on the BE behaviors of college students in Taiwan, this study has some limitations. First, as a cross-sectional survey, the study adopted a correlational design, which could only predict and discuss BE behaviors and associated factors rather than establish a causal relationship between them. Second, the study collected the participants’ perceived experience of BE via a self-administered questionnaire—the BES. Although its usage has prevailed in general populations as well as college students for BE behavior screening, a clinical diagnosis would be required to exclude other forms of eating disorders. Third, most participants came from universities in metropolitan areas, which presented relatively high academic and life stress. Fourth, all participants were overweight and obese college students, which could affect the implications of the results for other populations. Fifth, BE is usually caused by psychological and environmental factors. However, its relationship with biological factors such as family history of obesity and hormones should be considered. We recommend that further investigations and examinations be carried out in future studies. Sixth, some of the research variables were based on the literature; we recommend that relevant tools be used for evaluation in future studies.

## 5. Conclusions

Owing to the high prevalence of obesity worldwide, the problem of BE has drawn increasing attention in Asia. This study is one of the few to investigate the incidence of BE among college students in Taiwan, the results of which could not only enhance clinical empirical data but also help identify BE behaviors at an early stage and help campus mental health personnel provide care sooner. The self-administered BES adopted in this study not only saved time, budget, and manpower in data collection but also screened the severity level of BE behaviors, in turn facilitating early intervention by campus mental health personnel. Finally, this study found that the severity and probability of BE behaviors are significantly correlated with demographic data, such as sex and BMI, and psychosocial data including uncontrolled eating, weight loss diets, academic stress, peer competition, interpersonal distress, and unpleasant or major life events. Therefore, in addition to a comprehensive investigation of BE prevalence on the college campus, it is recommended that future research conducts in-depth investigations of binge eaters’ psychological distress and emotional regulation to gain a better understanding of BE-related concepts and develop effective intervention measures.

## Figures and Tables

**Table 1:** Demographic and psychosocial variables (n = 300).

Variable	Mean	SD	Number (n)	Percentage (%)
Age (18–24 years)	20.37	1.31		
Height (148–190 cm)	164.52	7.97		
Weight (55–170 kg)	81.08	16.20		
BMI (24.01–49.67 kg/m^2^)	29.82	4.63		
Sex				
Female			215	71.7
Male			85	28.3
Experience/feelings of previous events				
Uncontrolled eating				
Yes			46	15.3
No			254	84.7
Weight loss diet				
Yes			150	50.0
No			150	50.0
Being ridiculed for body shape				
Yes			152	50.7
No			148	49.3
Academic stress				
Yes			166	55.3
No			134	44.7
Peer competition				
Yes			95	31.7
No			205	68.3
Family pressure				
Yes			76	25.3
No			224	74.7
Interpersonal distress				
Yes			72	24.0
No			228	76.0
Unpleasant/major life events				
Yes			s68	22.7
No			232	77.3
BES score (17~34)	10.67	6.66		
No–mild (≤17)			248	82.7
Moderate (18–26)			46	15.3
Severe (≥27)			5	2.0

Note: BMI = Body Mass Index, BES = Binge Eating Scale.

**Table 2:** Distribution of responses to the Binge Eating Scale (n = 300).

Questions	Score of Severity and Its Distribution (%)			
	0	1	2	3
Q1. I am self-conscious about my appearance and weight	62.3	31.3	-	6.3
Q2. I tend to eat quickly and will feel uncomfortably full afterward.	24.3	32.7	33.7	9.3
Q3. I feel incapable of controlling my desire to eat.	67.3	25.7	-	7.0
Q4. I have a regular habit of eating when I’m bored.	93.0	-	7.0	
Q5. I feel like eating even when I am not hungry.	39.0	55.0	5.3	0.7
Q6. I feel guilty after overeating.	32.3	61.0	-	6.7
Q7. I lose control of my eating when dieting.	61.7	-	18.0	20.3
Q8. I frequently eat too much.	31.7	35.0	31.0	2.3
Q9. My calorie intake fluctuates significantly.	32.3	45.7	20.7	1.3
Q10. I feel incapable of controlling my urge to eat.	65.3	19.3	13.7	1.7
Q11. I cannot stop eating even when I am full.	42.3	54.3	3.0	0.3
Q12. I feel embarrassed about overeating in front of others.	64.0	26.7	7.0	2.3
Q13. I normally snack between meals.	90.7	-	6.3	3.0
Q14. My mind is often occupied by the desire to control my eating.	36.3	39.0	19.3	5.3
Q15. I have strong cravings for food.	47.0	44.0	3.7	5.3
Q16. I usually know whether I’m physically hungry and take the right portion of food.	63.3	29.3	7.3	-

Binge eating severity: 0 = no problem; 1 = mild; 2 = moderate; 3 = severe. - = scores collapsed into 3 or fewer groups on Q1, Q3, Q4, Q7, and Q13. Only three scores are available for Q6 and Q16.

**Table 3:** OR of logistic regression analysis predicting risk of BE behavior.

Variables	B	OR	*p* Value	95% CI	
				Lower	Upper
BMI	0.05	1.05	0.10	0.99	1.12
Sex	0.05	1.05 **	0.02	1.01	1.09
Uncontrolled eating	1.80	6.04 **	0.00	3.03	12.06
Weight loss diets	0.97	2.64 **	0.00	1.40	5.01
Being ridiculed for body shape	0.44	1.55	0.16	0.84	2.84
Academic stress	0.51	1.66	0.11	0.89	3.10
Peer competition	0.95	2.59 **	0.00	1.41	4.78
Interpersonal distress	0.75	2.12 *	0.02	1.12	4.02
Unpleasant or major life events	0.40	1.49	0.24	0.76	2.92

Note: Logistic regression results. Only variables derived from predictive factors with a significance * *p* < 0.05 and **p < 0.01 in the bivariate analysis were included. CI = confidence interval, OR = odds ratio. Cox and Snell R Square = 0.10, Nagelkerke R Square = 0.18.

## Data Availability

The datasets used and/or analyzed during the current study are available from the corresponding author (H.L.) upon reasonable request.
